# A Refractory Case of Amiodarone Thyrotoxicosis

**DOI:** 10.7759/cureus.28527

**Published:** 2022-08-29

**Authors:** Marta Fonseca, Mário Ferreira, Joana Paulo, Zélia Neves

**Affiliations:** 1 Internal Medicine, Hospital Professor Doutor Fernando Fonseca, Lisbon, PRT

**Keywords:** toxicity, adverse effects, thyrotoxycosis, atrial fibrillation, amiodarone

## Abstract

Amiodarone is frequently used to control cardiac arrhythmias, like atrial fibrillation. Despite its benefits, it has many adverse effects, particularly on the thyroid gland. We describe the case of a patient treated with amiodarone for paroxysmal atrial fibrillation, admitted to the emergency room with atrial fibrillation with a rapid ventricular response. Type II thyrotoxicosis was identified as the cause of the refractory arrhythmia. Since its refractoriness to both pharmacological and electrical therapy, there was a need to proceed with plasmapheresis and total thyroidectomy for hormonal and cardiac rhythm control. Therefore, it is essential to monitor the toxicity of amiodarone, a drug that can have both beneficial and devastating effects.

## Introduction

Amiodarone is an antiarrhythmic class III drug effective in several cardiac dysrhythmias control. For this reason, it is widely used. Despite its benefits, long-term use of oral amiodarone has been associated with several serious adverse effects like lung, heart, and ocular toxicity, but also less severe consequences such as liver enzyme elevation, neurologic dysfunction, or gastrointestinal disturbances [1].

Since it is composed of iodine, daily recommended doses of this element are sometimes exceeded [2]. The adverse effects on the thyroid gland are related to the excess iodine content and also the direct toxic effect [3]. These can translate into hypothyroidism or thyrotoxicosis. Amiodarone thyrotoxicosis is classified as type I if related to the increase of hormonal production (T3 and T4) as a consequence of iodine excess affecting a previously sick thyroid gland, and type II if it is a consequence of the direct toxic effect of the drug on a healthy gland, without an increase in hormonal production [2,4]. There can also be mixed forms, which can be more difficult to diagnose and treat [5]. Clinical manifestations of amiodarone thyrotoxicosis can be subtle and delayed, given its beta-blocker effect [6]. When they occur, it usually manifests as arrhythmia decompensation, with or without congestive heart failure, and classic symptoms of hyperthyroidism, such as weight loss, agitation, and low-grade fever [3].

Type I thyrotoxicosis treatment is based on thiamazole thirty to forty mg per day. Some patients require maintenance with a low dose for prolonged periods [7]. Concerning type II, treatment is based on corticoid therapy with prednisolone forty to sixty mg per day with or without associated anti-thyroid drugs, given its lower effectiveness in this type of disease and, eventually, amiodarone withdrawal [5,8]. Thyroidectomy is recommended in cases of refractoriness or intolerance to medical therapy, cardiac function deterioration, or severe underlying cardiac diseases. In those cases, thyroidectomy is a definitive therapy, and it seems to be safe to restart amiodarone if needed [9].

## Case presentation

We present a case of a 70-years-old female patient with a personal history of paroxysmal atrial fibrillation, type 2 diabetes mellitus, dyslipidemia, high blood pressure, and nephrolithiasis. She was chronically medicated with amiodarone, rivaroxaban, metformin, sitagliptin, gliclazide, atorvastatin, omeprazole, irbesartan, hydrochlorothiazide, amlodipine, furosemide, gabapentin. She had no known drug allergies.

**Figure 1:**
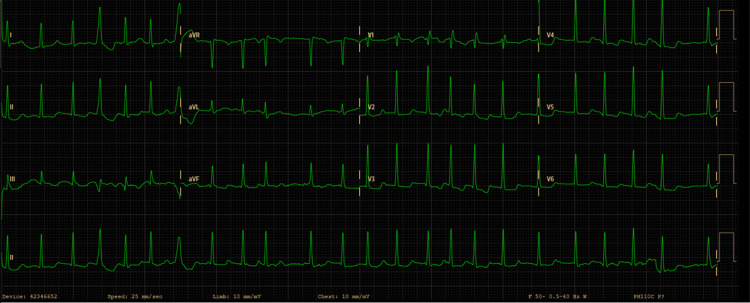
Admission ECG showing atrial fibrillation with rapid ventricular response

The patient presented to the emergency room with a three-day history of progressive worsening heart palpitation, dizziness, and weakness. On admission, the physical examination revealed a heart rate (HR) of 145 beats per minute (bpm), stable blood pressure (171/91 mmHg), without typical signs of heart failure, like inspiratory crackles on pulmonary auscultation, jugular vein distention or peripheral edema. The patient didn't present shaking, agitation, sweating, skin changes, or fever, and she denied appetite increase, weight loss, insomnia, increased heat, or any other changes in her usual health condition. The electrocardiogram (ECG, Figure 1) showed atrial fibrillation with a rapid ventricular response (A-fib RVR).

After a failed first attempt of rhythm control with bisoprolol, amiodarone, and digoxin, the subsequent approach included the use of intravenous propranolol and dual therapy with bisoprolol plus continuous infusion of amiodarone for less than 24 hours. Despite those efforts, tachyarrhythmia persisted (HR of 110-130 bpm). The patient was never hypotensive, and she was no longer complaining of palpitations and weakness; therefore, it was decided not to perform electric cardioversion. She was admitted to the intermediate care unit of the internal medicine department.

Later, the initial workup results (Table1) for refractory arrhythmia revealed abnormal thyroid function tests with thyrotoxicosis with free T4 (FT4) >7.77 ng/dL (reference value [RF] of 0.93-1.7 pg/mL), free T3 (FT3) 23.90 pg/dL (RF of 2-4.4 pg/mL) and thyroid stimulating hormone (TSH) below the measurable threshold (RF of 0.27-4.2 mUI/L). With these results, and without other relevant changes, hyperthyroidism was considered the cause of decompensation. The thyroid auto-immunity study was negative (anti-thyroglobulin, anti-myeloperoxidase, anti-TSH receptor autoantibodies), and the gland ultrasound was normal.

**Table 1:** Thyroid hormone levels evolution throughout hospital admission and four months after discharge FT4 - free T4; FT3 - free T3; TSH - thyroid-stimulating hormone; RF - reference value

	At admission	Week five	Discharge	Four months after discharge
FT4 (RF of 0.93-1.7pg/mL)	>7.77	>7.77	1.02	1.96
FT3 (RF of 2-4.4pg/mL)	23.90	3.73	0.92	2.53
TSH (RF 0.27-4.2mUI/L)	<0.005	<0.005	0.355	1.97

We assumed the diagnosis of Type II thyrotoxicosis secondary to chronic amiodarone therapy, which the patient had been under for the last two years. The diagnosis and initiation of treatment were made by a private cardiologist. The patient lost follow-up by the doctor but kept the initial medication.

Amiodarone was discontinued, and she began treatment with prednisolone 40 mg per day and thiamazole 30 mg per day. There were no other signs of amiodarone toxicity. After the beginning of therapy, the patient's heart rate was kept above 120 bpm, with normal blood pressure and no associated symptoms. Since the patient was already under anticoagulation therapy at home, it was decided to perform electric synchronized cardioversion as an attempt to rhythm control, despite toxic hormone levels. Sinus rhythm (SR) was achieved with a heart rate of 80 bpm after one single 120J shock. Besides a successful initial result, after a few days in SR, the patient returned to A-Fib with tachycardia, remaining normotensive. It was decided to keep the pharmacological approach with propranolol and digoxin since the patient kept stable blood pressure and hormone levels were still at toxic values and under direct therapy.

After five weeks under high doses of thiamazole and prednisolone, with thyroid hormone dosing still on toxic levels and cardiac rhythm as A-fib RVR, the patient started showing adverse effects of prolonged corticosteroid therapy, namely a difficult to control diabetes mellitus and corticosteroid-induced myopathy. Concurrently she developed heart failure signs (with inspiratory crackles) and a slight decrease in systolic function on echocardiogram (with an ejection fraction of 45-50%).

Thyrotoxicosis was therefore classified as refractory to pharmacological therapy. Three sessions of plasmapheresis were performed, followed by a radical thyroidectomy. The surgical intervention and postoperative period elapsed without problems. After surgery, the patient started hormone replacement therapy with levothyroxine, with the need for small dose adjustments. The heart rate was also controlled with bisoprolol 2.5 mg per day, although the patient remained in A-fib. An echocardiogram at discharge time showed normal systolic function, mild mitral valve insufficiency, and mild left atria enlargement (which was assumed as the probable cause for chronic A-fib).

## Discussion

Amiodarone was the chosen antiarrhythmic drug for A-fib control in this patient two years before this event. It is described as the most effective antiarrhythmic drug for A-fib, but its common side effects may obligate to discontinuation of the drug [10]. When used, toxicity should be monitored very closely. In this case, the fact that the patient lost regular follow-up by her doctor might have contributed to the long-term use of the drug without monitoring of side effects and the development of the described episode.

Type II thyrotoxicosis, due to amiodarone's direct toxic effect on thyroid cells, can develop years after the beginning of the drug, and hyperthyroidism symptoms can persist for months to years and can, eventually, evolve to transitory hypothyroidism [5,11]. Usually, thyroid function disorders related to amiodarone use are less severe and reversible in a few weeks after starting pharmacological therapy. Thyroidectomy is needed for a small portion of cases [12]. However, in this case, even after several attempts for over a month, the patient had a negative clinical progression, with maintained hyperthyroidism, tachydysrhythmia, and signs of heart failure. Furthermore, she developed adverse effects from corticoid therapy. For this reason, there was a need for thyroidectomy to control the toxic effects of amiodarone. To decrease the potential surgical risks of toxic thyroid hormone levels, it was decided to perform plasmapheresis before thyroidectomy.

## Conclusions

This case illustrates the potentially severe consequences of a drug and the importance of close surveillance of its side effects in several organs. Although amiodarone has several proven benefits, its use has some risks that can never be forgotten and must be actively investigated. Furthermore, this case report also demonstrates the importance of drug toxicity in investigating some disorders' etiology. 
